# The body and the fading away of abstract concepts and words: a sign language analysis

**DOI:** 10.3389/fpsyg.2014.00811

**Published:** 2014-07-29

**Authors:** Anna M. Borghi, Olga Capirci, Gabriele Gianfreda, Virginia Volterra

**Affiliations:** ^1^Department of Psychology, University of Bologna and Institute of Cognitive Sciences and Technologies, Italian National Research CouncilRome, Italy; ^2^Institute of Cognitive Sciences and Technologies, Italian National Research CouncilRome, Italy; ^3^National Institute for the DeafRome, Italy

**Keywords:** abstract concepts, abstract words, Italian Sign Language (LIS), sign languages, embodied cognition, metaphor, signs, iconicity

## Abstract

One of the most important challenges for embodied and grounded theories of cognition concerns the representation of abstract concepts, such as “freedom.” Many embodied theories of abstract concepts have been proposed. Some proposals stress the similarities between concrete and abstract concepts showing that they are both grounded in perception and action system while other emphasize their difference favoring a multiple representation view. An influential view proposes that abstract concepts are mapped to concrete ones through metaphors. Furthermore, some theories underline the fact that abstract concepts are grounded in specific contents, as situations, introspective states, emotions. These approaches are not necessarily mutually exclusive, since it is possible that they can account for different subsets of abstract concepts and words. One novel and fruitful way to understand the way in which abstract concepts are represented is to analyze how sign languages encode concepts into signs. In the present paper we will discuss these theoretical issues mostly relying on examples taken from Italian Sign Language (LIS, Lingua dei Segni Italiana), the visual-gestural language used within the Italian Deaf community. We will verify whether and to what extent LIS signs provide evidence favoring the different theories of abstract concepts. In analyzing signs we will distinguish between direct forms of involvement of the body and forms in which concepts are grounded differently, for example relying on linguistic experience. In dealing with the LIS evidence, we will consider the possibility that different abstract concepts are represented using different levels of embodiment. The collected evidence will help us to discuss whether a unitary embodied theory of abstract concepts is possible or whether the different theoretical proposals can account for different aspects of their representation.

## Introduction

To what extent are cognitive capacities learnt through action? According to embodied and grounded views, acting and interacting with the objects and the physical and social entities present in the environment represent the basis of our cognitive abilities (e.g., Wilson, 2002). Research on embodied and grounded cognition has rapidly grown in the last 10–15 years, as widely acknowledged by different scholars (e.g., Chatterjee, 2010; Gentner, 2010; for a review see Borghi and Caruana, in press).

In the last years, much behavioral and neuroscience evidence has been provided, showing that concepts and language are grounded on perception and action systems (for reviews, see Gallese and Lakoff, 2005; Barsalou, 2008; Fischer and Zwaan, 2008; Gallese, 2008; Jirak et al., 2010; Meteyard et al., 2012; for special issues, see Borghi and Pecher, 2011). However, the perspective of embodied and grounded cognition is confronted with some unsolved issues and open challenges. One of the major challenges is represented by the possibility to account for the representation of abstract concepts and words meanings (see the recent special issue by Tomasino and Rumiati, 2013). With “abstract words meanings” we intend the meaning of words such as “philosophy” and “truth,” that apparently do not have a single, easily identifiable, imaginable and concrete referent. Their referents are instead situations, events, mental states, conditions. Specifically, whether the embodied account holds only for concrete concepts and words or whether it can be extended to abstract concepts and words as well is still a matter of debate. A number of scholars have argued that, while embodied theories are able to account for words referring to concrete objects (e.g., bottle), supported by convincing evidence, the story is completely different if we consider the domain of abstract words, due both to theoretical limits and to the lack of compelling empirical evidence (e.g., Dove, 2009, 2011).

Our paper deals with abstract concepts representation. First, we will consider the possibility that different degrees of embodiment are involved in the representation of concrete and abstract concepts. Second, we will verify whether different abstract concepts are represented using different levels of embodiment. We will distinguish between direct forms of involvement of the body and forms in which concepts are grounded differently, for example relying on linguistic experience. To handle these theoretical issues in the present paper we will first provide a brief outline of the major recent accounts of abstract concepts within embodied and grounded theories (for recent reviews see Pecher et al., 2011; Borghi and Binkofski, 2014). The embodied cognition perspective has indeed developed different proposals that attempt to explain abstract concepts representation.

The novelty of the present contribution is that we will verify the solidity of these theories in light of examples taken from one of the many Sign Languages (from now on SL): the Italian Sign Language (from now on LIS, Lingua dei Segni Italiana), the language used within the Italian Deaf community, described and analyzed since about 30 years.

### Definition

Defining abstract concepts and words is not an easy task. It is noteworthy that the term “abstract” is represented in LIS by a sign located near the head and referring to something that cannot be touched and grasped, to something that is not material and concrete but that rather fades away.

Here we will adopt a rather broad operational definition of abstract terms. We define abstract the words and the signs that, differently from concrete ones, do not refer to single, concrete and manipulable items, but are rather grounded in situations, events, mental states, etc. Abstract words are typically rated as less imaginable as concrete ones, they are more complex than concrete words since they often refer to relations between elements rather than to single objects/entities, and they are characterized by higher intersubjective and intra-subjective variability (see Borghi and Binkofski, 2014, for clarifications on this definition). Notice however that the opposition between concrete and abstract concepts might not be a dichotomy but rather a continuum. Ratings asking people to judge the concreteness of large sets of words showed that concrete and abstract concepts are distributed in a bimodal way, falling into two big clusters (according to features, such as tangibility or visibility); within each cluster, however, the entities had different concreteness degrees (Nelson and Schreiber, 1992; Wiemer-Hastings et al., 2001).

Despite the difficulty in finding a shared definition, embodied theories of abstract concepts are numerous; below we will briefly illustrate the most important ones.

### Main embodied theories of abstract words

According to classical Embodied Cognition (EC) theories of abstract words there would not be a substantial difference between concrete and abstract words, since both are grounded in perception, action and emotional systems. For example, the abstract concepts of number would be grounded in action due to finger counting experience (for a review, see Fischer and Brugger, 2011). Further evidence in support of this view is obtained by studies that link words to action, for example by evidence on the Action-sentence Compatibility Effect (ACE). Results showed that judging the sensibility of sentences which describe the transfer of both concrete objects and abstract information (e.g., “giving the pizza” vs. “giving the information”) requires less time when the action implied by the sentence matches the action required to make the response (Glenberg and Kaschak, 2002; Glenberg et al., 2008a,b). This finding suggests that the mechanisms underlying transfer of abstract concepts (e.g., “the information”) are the same as those underlying transfer of concrete ones (e.g., “the pizza”) (see also Guan et al., 2013).

The other EC theories we will illustrate posit that abstract and concrete concepts and words are represented differently. The most influential one is probably the Conceptual Metaphor Theory, which states that abstract concepts are represented by image schemas derived from concrete domains. Evidence supporting this theory has shown for example that similarity is represented as closeness, categories as containers, and that the abstract notion of time is mapped onto the concrete domain of space (e.g., Lakoff and Johnson, 1980; Gibbs and Steen, 1999; Boroditsky and Ramscar, 2002; Casasanto and Boroditsky, 2008; Boot and Pecher, 2010, 2011; Casasanto et al., 2010; Flusberg et al., 2010; Lai and Boroditsky, 2013).

Further theories identify differences in content between concrete and abstract concepts. According to Barsalou and Wiemer-Hastings (Barsalou, 1999; Barsalou and Wiemer-Hastings, 2005), abstract concepts differ from concrete concepts as the first activate situations and introspective relationships more frequently. Evidence in favor of this approach is based mainly on results of feature generation tasks, showing that, whereas with concrete concepts, such as “bottle,” people tend to produce mostly properties referring to perceptual characteristics such as color, size, shape, matter, parts (e.g., “green,” “plastic,” “neck”), abstract concepts such as “freedom” evoke more frequently situations, events, introspective states (e.g., “running on the grass,” “exiting from prison,” etc.).

A novel proposal advanced by Vigliocco and colleagues (Kousta et al., 2011; Vigliocco et al., 2014) states that abstract concepts differ from concrete ones in content, since they rely more on emotional experience. Analyzing a large database Kousta et al. (2011) demonstrated that, when imageability was kept constant, emotional valence was a significant predictor of concreteness ratings. Recent brain imaging evidence (Vigliocco et al., 2014) further supports this view.

Other recent approaches, such as the Language and Situated Simulation Theory (LASS) (Barsalou et al., 2008; Simmons et al., 2008), the Symbol Interdependence Theory (Louwerse and Connell, 2011), the proposal by Dove (2011, 2014) and the Words As social Tools (WAT) proposals (Borghi and Cimatti, 2009; Borghi, 2014; Borghi and Binkofski, 2014; evidence in Borghi et al., 2011; Scorolli et al., 2011, 2012; Sakreida et al., 2013), argue that both linguistic and sensorimotor information are crucial for conceptual representation. LASS does not specifically focus on abstract concepts, but on conceptual representation more generally. According to LASS, both the linguistic and the simulation system are activated during conceptual processing; the linguistic system is faster and more superficial, while the simulation system is engaged for understanding of meaning. In some situations using the linguistic system represents a shortcut as it allows to respond immediately to a task (particularly to linguistic tasks) without necessarily accessing to conceptual meaning (Pecher and Boot, 2011). In a similar vein, Louwerse's Symbol Interdependency Theory states that shallow linguistic representations precede deeper perceptual representations (Louwerse, 2011; Louwerse and Connell, 2011; Connell and Lynott, 2012).

Compared to the other multiple representation theories, WAT (Borghi and Cimatti, 2009; Borghi and Binkofski, 2014) and Dove's view (Dove, 2014) focus specifically on the difference between concrete and abstract concepts and words. According to both views abstract concepts representation relies more on language than representation of concrete words. In his proposal on abstract concepts Dove (2011, 2014) stresses the important scaffolding role language can play and the fact that the abilities acquired thanks to language allow its use not only as a means of communication but of thought as well. The main tenets of WAT are the following: a. both concrete and abstract concepts are embodied and grounded in perception and action systems, b. for abstract concepts linguistic information plays a more crucial role than for concrete ones, c. this is due to the different acquisition modality of concrete and abstract words; d. this distributional difference is reflected in the representation in the brain of concrete and abstract concepts, e. given that representation of abstract concepts is more influenced by language, linguistic diversity has a major impact on abstract concepts representation. An important principle of the WAT proposal concerns the acquisition mechanism of the two kinds of words: with concrete words, the concrete entities (e.g., book) can be perceived together with their linguist labels. In the case of abstract words, the linguistic experience might be more important, because typically abstract words do not have a single concrete referent and also because they usually refer to exemplars differing to a great extent. Verbal labels are hence used to assemble a set of quite sparse and diverse sensorimotor experiences (e.g., we probably put together different experiences of freedom once we have learned the word “freedom”). Evidence in support of this proposal is multifaceted (for review see Borghi and Binkofski, 2014). Brain imaging studies demonstrated greater engagement of the verbal system for processing of abstract concepts and greater engagement of the perceptual and motor system for concrete concepts (e.g., Binder et al., 2005; Sabsevitz et al., 2005; Rüschemeyer et al., 2007; Desai et al., 2010; Sakreida et al., 2013), and behavioral research has shown a high cross-linguistic variability with abstract words (e.g., Boroditsky, 2011). Notably, acquisition evidence has shown that the process of acquisition of the two kinds of words might differ (e.g., Wauters et al., 2003; Borghi et al., 2011). In particular, studies on Mode of Acquisition (MOA) (e.g., Wauters et al., 2003) have shown that children acquire the meaning of concrete words, such as “bottle,” associating the word with its referent, the bottle, or with an action typically performed with or on the bottle by themselves or by another individual (Capirci et al., 2005). The meaning of abstract words like “grammar” or “philosophy,” instead, has to be explained by means of language. Finally, the meaning of a word like “tundra” can be acquired in both ways, depending on the environment where it is learned. MOA ratings, which correlate but are not totally explained by age of acquisition, concreteness and imageability, gradually change with age: initially acquisition is mainly perceptual, later it is mainly linguistic.

### The challenge

The question theorists adopting an EC approach have to ask is the following: is it possible to account for abstract words with a unified framework? Isn't it possible, instead, that the domain of abstract words is not homogeneous, and that the different subsets of abstract words have to be explained relying on different mechanisms? Recent studies showing fine-grained differences between subsets of abstract words (e.g., Ghio et al., 2013; Roversi et al., 2013) suggest that this might be the case. For example, abstract words as diverse as “category,” “truth,” and “risk” could rely on different mechanisms: the first could metaphorically evoke a container (Boot and Pecher, 2010), the second could evoke linguistic information and the third might activate situations. If this is true, this would lead us to abandon the overall notion of abstractness and to partition the domain into sub-domains of abstract words.

One intriguing way to understand the way in which abstract words are represented and to deal with the challenge abstract words pose to the EC perspective is to analyze how they are dealt with in sign languages. In our opinion, the way in which sign languages encode concepts into signs can help us understand how abstract linguistic items are represented, and which theory among those on abstract concepts can better account for their meaning.

Linguistic research undertaken since Stokoe's (1960) seminal work on American Sign Language (ASL) has led to the discovery and description of a very large number of national sign languages, now widely recognized by the scientific community as full-fledged, natural languages, which include Italian Sign Language or LIS (Volterra, 1987; Pizzuto and Corazza, 1996). In the last edition of the Ethnolog database 137 Sign Languages (SL) are listed. It has been shown that, even though these languages are perceived and produced in the visual-gestural (rather than in the vocal-auditory) modality, they satisfy the communicative and expressive needs of a community and possess all the basic linguistic components including phonological, lexical, syntactic and grammatical systems. Just as words of a spoken language are formed on the basis of phonemes in various combinations, all signs of a signed language are formed by combining a defined number of formational parameters (called also as cheremes). More precisely, a sign can be broken down into four basic parameters: the form or configuration taken on by the hand; the orientation the hand takes on while making the sign; the location in which the sign is performed; the movement the hand describes.

As Penny Boyes Braem pointed out already in 1981, signed lexical units are often made up of formal features visually motivated and thereby iconic. Their visual motivation is not idiosyncratic, it derives from regularities at the level of formational parameters. Handshapes, for example, are often linked to features of a sign's meaning via reference to some peculiar visual forms (Pizzuto et al., 1995; Pietrandrea and Russo, 2007). The same holds true for location and often for movement (for a comprehensive analysis of the iconicity of the LIS parameters, see Pietrandrea, 2002). In spite and beyond important structural resemblances between Sign Languages and Vocal Languages, equally relevant structural differences need to be taken in due account (Sutton-Spence, 2005; Cuxac and Sallandre, 2007; Pizzuto et al., 2007; Perniss et al., 2010; West and Sutton-Spence, 2010; Boyes Braem et al., 2012; Meurant et al., 2013; Perniss and Vigliocco, 2014). The grammar and the syntax of a sign language are expressed in various ways, including use of space, modulation of movement, facial expression and position of the trunk and shoulders. A great deal of research has been carried out on the signs used by the Deaf Italian community (a complete bibliography on LIS is available at biblioLIS http://www.istc.cnr.it/sites/default/files/u182/bibliolis_arg_2011.pdf).

To our knowledge the relationship between sign languages and abstract concepts has been investigated in a few studies so far (e.g., West and Sutton-Spence, 2010). In 2005 the Journal “Sign Language Studies” devoted a Special Issue to a crosslinguistic analysis of SL in the metaphorical domains of thought and communication. Linguists studying different sign languages (British, American, Catalan, and Italian) examined the mappings involved in SL metaphors, showing the process of embodiment active in metaphorical structures. Some structures share similarities across sign languages but there are also some interesting differences. Russo (2005) suggests that signed language metaphors are intrinsically related to aspects of the linguistic and cultural dimensions of a specific deaf community. More recently Roush (2011) has addressed the issue of the cognitive representation of abstract terms in sign languages. The author analyzed how a number of abstract words are represented in American Sign Language (ASL). Roush (2011) applied a specific linguistic-cognitive framework, the Conceptual Metaphor Theory, to investigate how the area of (im)politeness is conceptualized through metaphors and reflected and iconically represented in ASL. Our approach shares with Roush the view that using sign languages is an important perspective helping understand the way in which concepts are represented, however the ultimate aim why we use sign languages for investigating cognitive issues is slightly different. While Roush focuses on a specific theory we move from a variety of embodied theories struggling to account for abstract concepts representation. Specifically, our investigation is aimed at analyzing how abstract concepts belonging to different domains are represented in LIS, assuming that this analysis will allow us to understand whether the category of abstract terms is homogeneous or whether it needs to be re-organized into different sub-sets.

### Hypotheses

We advance the following hypotheses. First, in line with all embodied theories we predict that all the considered abstract concepts are at least in part grounded in the sensorimotor system. This guarantees the fact that the problem of symbol grounding (Harnad, 1990) is not present, since symbols used to represent abstract concepts are not arbitrarily linked to their referents.

At the same time, however, we predict that theories taking into account only sensorimotor nonlinguistic information will not be able to explain all examples we provide. In our view a unified framework, either based only on sensorimotor (for a review, see Pecher et al., 2011) or only on linguistic information (e.g., Paivio, 1986) will not be able to account for the differences between kinds of abstract concepts. In line with multiple representation theories we predict, instead, that to account for some abstract concepts a combination of sensorimotor, emotional, and linguistic information will be necessary. With “linguistic information” we intend any kind of exploitation of forms derived from any kind of language, be the same sign language or a different sign or spoken language. An example is the concept of “causation”: it is grounded in sensorimotor information since it might activate a variety of situations in which, for example, one element determines an effect on another one (e.g., a ball hurting another ball and provoking its movement, a handle being pressed to open a door etc.); at the same time, however, to acquire the concept children might rely on explanations of what causation is provided by others, such as parents or teachers, or by authoritative written sources, such as dictionaries, encyclopedias, etc. Another example highlighting how the formation of abstract concepts can rely on linguistic sources is the concept of “linguistics,” which originates from and refers to the more concrete concept of “language.” Specific examples pertaining SLs, such as linguistics, language, truth, etc., are discussed later in the paper. To highlight the role of linguistic information we have selected on purpose concepts where the role of linguistic elements is particularly evident, even if sensorimotor information still plays a role. This combination of sensorimotor and linguistic information is what we mean when we speak of “different levels of embodiment.”

## LIS evidence

In the present section we will provide novel evidence on LIS signs supporting the most important theories we have presented. The examples we are going to illustrate and discuss are mainly taken from a corpus collected by Gianfreda (2011; Gianfreda et al., 2014). The corpus was originally collected to explore the linguistic forms through which Italian Sign Language (LIS) signers realize communicative functions related to the expression of certainty and uncertainty, focusing on dimensions already explored for spoken Languages and for which theoretical constructs such as epistemic modality and evidentiality have been proposed. Conversations in LIS between deaf people communicating through a video-chat software have been collected and analyzed. In this type of interaction, the technological instrument itself permits to record the conversations in a less intrusive manner. Both participants are obliged to maintain themselves in front of the webcam and to optimize video quality in order to understand their sign language productions. The software automatically creates, in real time, two video windows for each interlocutor; through split-screen it is possible to analyze efficiently the synchronization between signs, facial expressions and body actions produced by both participants. Focusing on low-structured interactions we have been able to observe linguistic units typical of LIS as they spontaneously emerge in effective situations of language use.

The corpus consisted of six exchanges: four completely free and two on a suggested topic. The time duration range of conversations was from 23 to 51 min. Conversational exchanges in which signers were expressing certainty and/or uncertainty have been identified and transcribed through Sign Writing (SW: Sutton, 1999). SW is a system based on a set of “glyphs,” which, combined together in graphic units, permit to write or transcribe signs, allowing an external reader to reconstruct sign language forms. A textual qualitative analysis has been conducted to better identify and describe the linguistic forms used by the LIS signers.

All examples of signs provided and words reported in the present paper to support different theories of abstract concepts are selected from the corpus above described except for the last three LIS signs mentioned in the present paper: language/linguaggio, linguistics, and communication. Our analysis has obviously no pretense to be exhaustive. However, we believe that providing examples supporting or disconfirming a given theory is a useful strategy. Consider for example studies providing support to the Conceptual Metaphor Theory: in one study it is shown that similarity is conceived as spatial contiguity (Boot and Pecher, 2010), in another that category is intended in terms of container (Boot and Pecher, 2011), in many studies it is shown that the abstract notion of time is conceived in terms of the more concrete notion of space (e.g., Boroditsky and Ramscar, 2002; Casasanto, 2008). These examples provide support to the theory, even though they do not tell us that the theory is necessarily always true. At the same time, providing even one single example disconfirming a theory can widely limit its application range, or its generality. This is exactly the strategy we will follow in the present paper. In the present text signs are reported by English glosses and often by figures.^1^ A complete list of all the figures can be found in the supplementary materials.

Different signs can provide support for the Conceptual Metaphor Theory. Specifically, we will refer to examples that highlight the use of body parts in an iconic way to refer to underlying metaphors. These manual signs are executed in different iconically motivated body parts (e.g., eyes, head, chest).

Concrete examples are represented by the LIS signs glossed as see and hear. Both verbs refer to the acquisition of characteristics of external reality through the appropriate sensorial organs. The movement of the first sign starts from the eye toward the external space while the second sign is executed near the ear with a movement toward the body. Two further signs are executed in these face locations, i.e., perceive-through-sight and perceive-through-hearing.

**Figure 1:**
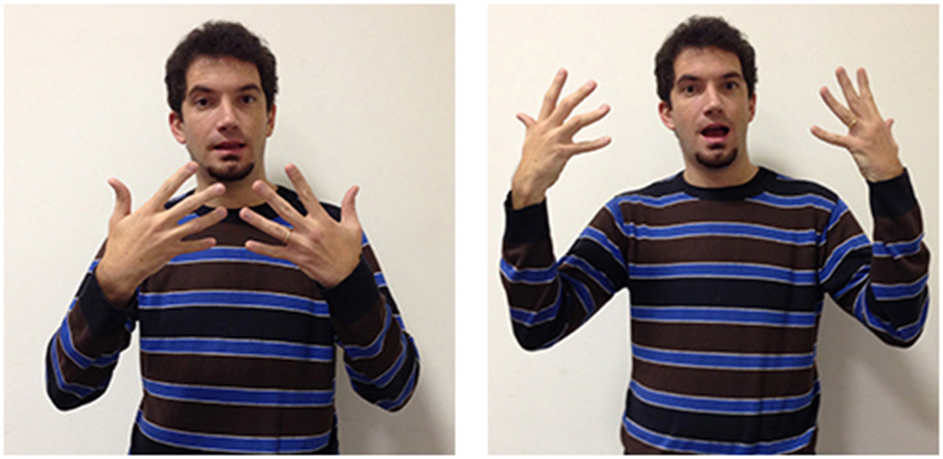
**LIS sign clear**.

These two signs share the same configuration and the same movement, but their different locations indicate the different sensorial modalities (sight and hearing) through which the perceptions occur. Notice that deaf people tend to exclude audition when they refer to perceptual activity in general since this modality is not very useful in their representation of the world. The verbs hear and perceive-through-hearing are strictly associated to experiences of hearing individuals. This aspect helps us understand why in LIS the notion knowing is seeing is more meaningful and therefore more used. Several metaphors rely on this concept and explain many LIS lexical units. For example in the sign clear (Figure 1) both hands are initially located in front of the eyes with hand configurations suggesting an initial partial obscurity. The two hands move laterally, away from the body, expressing broad, unimpeded perception. The same hand configuration is used for the sign seem, which is typically used to express something acquired through perception. The association between the perceived entity and its interpretation is uncertain (for the corresponding ASL sign, see Wilcox and Wilcox, 1995; Wilcox and Shaffer, 2006).

The location in which the sign seem is produced, i.e., the space between the forehead and the eyes, reflects perceptual and cognitive processes. The signer indicates that his/her epistemic belief concerning the content he/she is expressing is grounded on some kind of evidence, which should be further verified. The sign can be linked not only with inferences based on acquired evidence but also on memory retrieval. When the sign seem is produced with half-closed eyes, and sometimes also with tensed cheeks, it expresses a focusing process concerning perception or memory.

**Figure 2:**
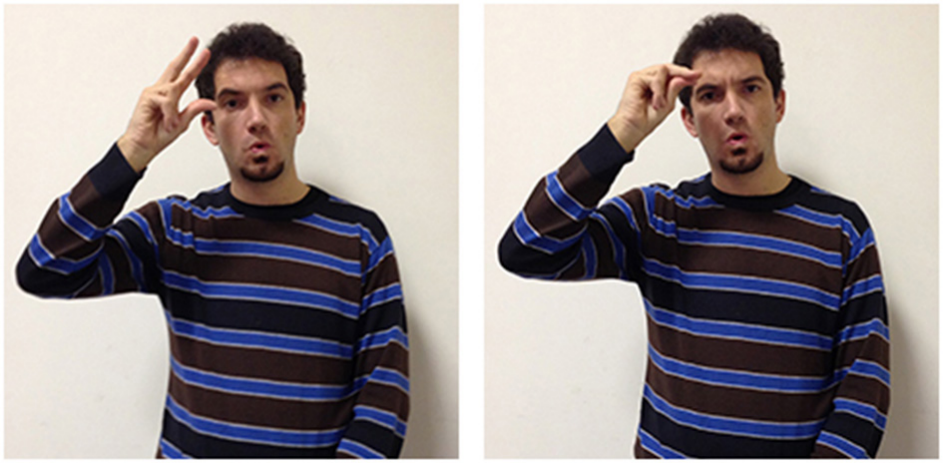
**LIS sign to know**.

Many verbs are produced around the forehead. For example, to learn, to know, to understand, to forget, to remember, and acknowledged all seem to link to the underlying metaphor of the head as the location of cognitive and memory activities. For the sign acknowledged, the signer first locates his/her index finger in the direction of the head; after this first movement a quick rotation of the wrist with the open hand follows, representing the sign translatable as finish, which allows indicating the completion of the action expressed from the main verb. The mental process is signaled in a slightly different way from the sign to know (Figure 2) in which the fingers thumb, index and medium, extended, quickly touch each other. In the sign to remember, instead, the index and medium finger, extended and joined, are placed on the forehead, suggesting that the remembered object is stably located within the head. Some of these verbs, also located around the forehead, i.e., to learn, to understand, and to forget, rely on the underlying metaphor of the mind as container: perceptual traces, recalls, linguistic information, conceptual nets are formed and stored in the head. Clearly present in the conceptual metaphor here is a movement toward or away from the head. One of the clearest examples is the sign to learn in which all the extended digits quickly touch each other and move toward the signer's forehead as if bringing in something from the external space (see Supplementary Materials). The same digit configuration, but with the palm of the hand orientated laterally to the head and combined with a repeated circular movement is found in the sign to think (see supplementary materials). The forehead location, symbolizing the place where the “objects” of perceptual, mnestic and cognitive processes can be seen and manipulated, explains the formation of many lexical units in a variety of sign languages (see Brennan, 2005; Jarque, 2005; Russo, 2005; Wilcox, 2005, 2007).

**Figure 3:**
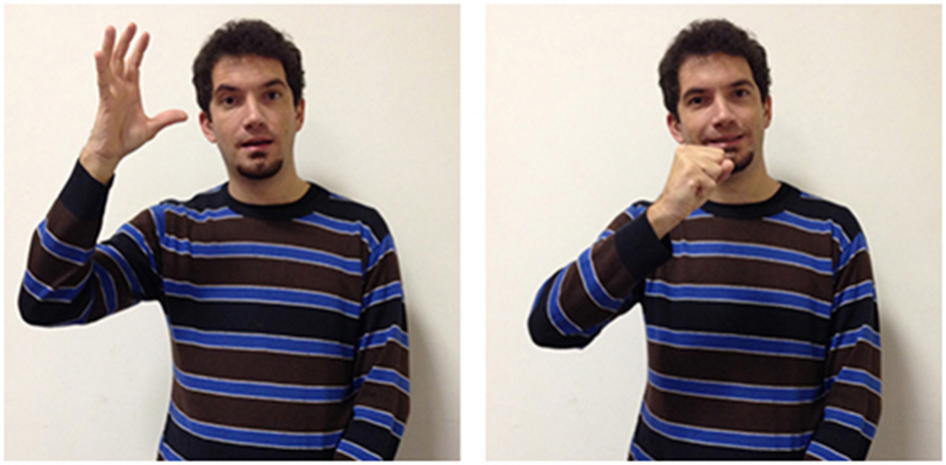
**LIS sign to understand**.

Another interesting example is the sign to understand (Figure 3), which uses the same movement found in LIS to indicate grasping of physical objects. The main difference between the signs to understand and to grasp is in their location: to grasp is located in the neutral space in front of the signer's chest, whereas to understand is produced near the signer's head; this clearly represents a form of metaphorical extension, as it suggests that understanding is grasping and putting something in the head-container (Russo, 2004). This metaphor reflects the Latin etymology of the word *com-prehendere*, which is maintained also in other European sign languages. In ASL, a different underlying metaphor is present: the concept to understand is conveyed through a fist-like handshape placed near the forehead from which the index finger is then extended, indicating the emergence of a thought-object from mental processes (Wilcox, 2005).

**Figure 4:**
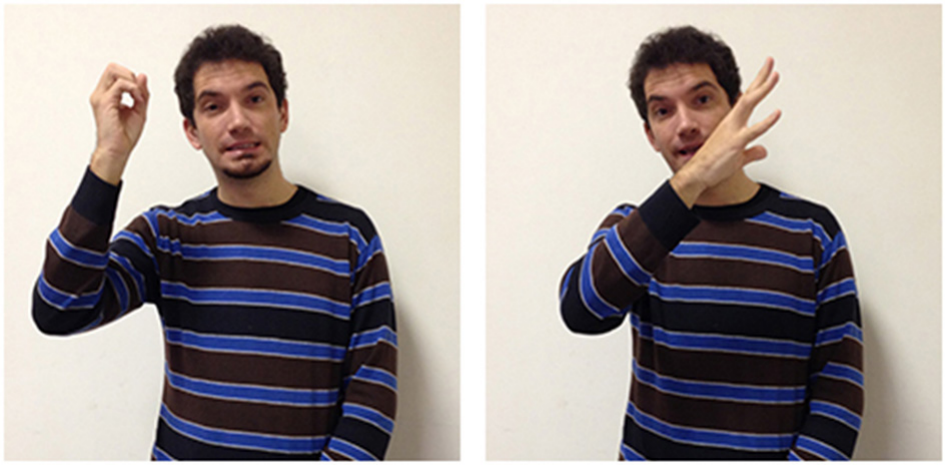
**LIS sign to forget**.

The metaphor of the head as container underlies also the LIS sign to forget (Figure 4), in which the closed hand moves to the other side of the head, symbolizing the sliding away of a mental object which had been previously “grasped” by the signer, and opens: the close hand indeed moves away from the head toward the lateral space.

The examples discussed so far support the idea that abstract terms are represented through conceptual metaphors. But some signs, such as to learn, to understand, to forget, also support the ACE view, as actions executed with physical objects are relevant for the representation of the concept expressed through the metaphor.

Other LIS signs expressing uncertainty are linked to a concrete physical object such as a balance.

**Figure 5:**
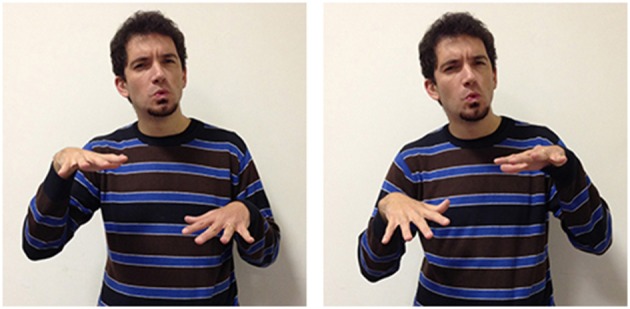
**LIS sign to doubt**.

In the LIS sign to doubt (Figure 5) the oscillating movement of the two hands with downward orientated palms expresses uncertainty. The ASL sign maybe looks very similar but the hand configuration differs, as the hand palms are oriented upwards, referring more explicitly to a balance with two similar weights, metaphorically extended to cognitive activity (Wilcox and Wilcox, 1995; Wilcox, 1996).

The LIS signs perhaps/maybe and about both have handshapes and locations which are very similar to that of to doubt, but differ in their movement of an oscillating wrist. These two signs occur, however, in different contexts, in which they are accompanied by different mouth^2^ patterns. perhaps tends to reinforce hypothetic statements, or to reduce the impact of the speaker's statements. about, instead, can be mostly found in expressions in which the signer defines numerical quantities or time periods, ascribing a character of approximation to the expressed values.

**Figure 6:**
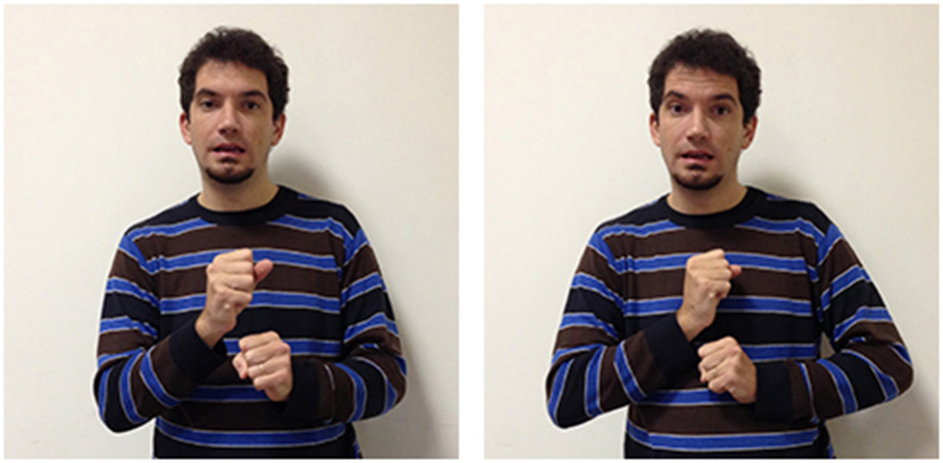
**LIS sign to believe**.

Other signs are executed on different body locations, which can also provide a motivation from an iconic point of view. For example many LIS signs executed on the chest are referring to feelings, such as love, hatred, rage. However, signs linked to mental activity can also be produced near the chest. For example, the sign to believe (Figure 6) is made with the upper side of the two fists touching the heart; in LIS this sign can also mean trust.

A sign that specifically supports the ACE view is to constrain. In this “agreement verb,” the hand (thumb and index finger bent as if to grasp a small object) can move toward the signer's neck or with reversed palm orientation move toward another point in space. This change in palm orientation and movement direction specifies the arguments of the verb (“x is constrained by y,” “x constrains y”). The underlying metaphor is clearly linked to the expression “Grab somebody by the throat.”

**Figure 7:**
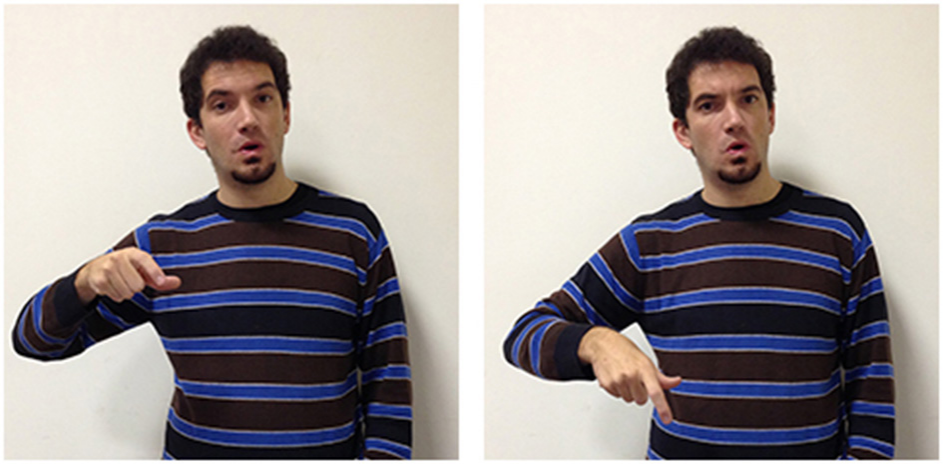
**LIS sign by force**.

A more abstract version of this sign is made in neutral space, with a sharp downward wrist flexion. This version of the sign is glossed as by force (Figure 7). In this sign the constraining agent is less salient or completely absent and the sign refers to actions where a norm should be applied. It is often used with an epistemic value: to ascertain that the described facts are as they should be, or that given qualities or actions are necessary to realize or accomplish a given state of affairs. Another LIS sign directed toward the speaker's neck expresses the signer's obligation but with a different hand configuration (bent V). This sign (to be constrained) expresses an obligation not determined by an agent but by the external events.

Evidence favoring the theory that emotions characterize abstract concept representation (Kousta et al., 2011) can be found not only in the LIS sign to believe discussed previously, but also in the sign to express oneself (see Supplementary Materials). In this sign, the two hands move up and outward in an arc from the chest toward external space, opening to a spread “5 handshape,” an action resembling the way in which we throw objects out of a container. It might not be necessarily obvious how these two concepts imply emotional components; however, as clarified in the introduction, according to the view proposed by Kousta et al. (2011) and Vigliocco et al. (2014) view all abstract concepts have emotional components, even if in different degrees. Compared to the head, the chest activates more general metaphors, linked not only to cognitive aspects but to emotional elements as well.

The specific metaphors underlying the signs often reflect cultural differences. For example, in Japanese Sign Language, signs related to thinking are executed in the area surrounding the chest (Wilcox, 2005). In Catalan Sign Language (LSC) ideas can be conceived as having liquid form and the results of learning process can be shown as a liquid contained in the learners' lower torso (Jarque, 2005).

**Figure 8:**
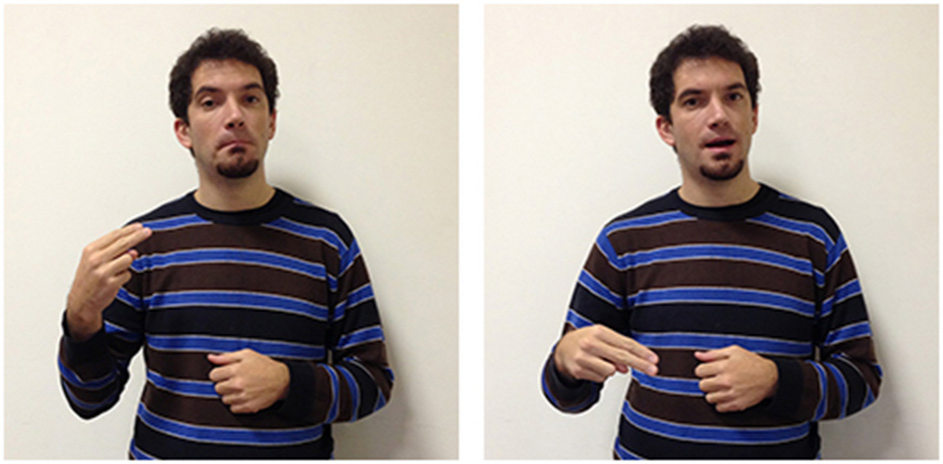
**LIS sign impossible_h-pa-pa_**.

**Figure 9:**
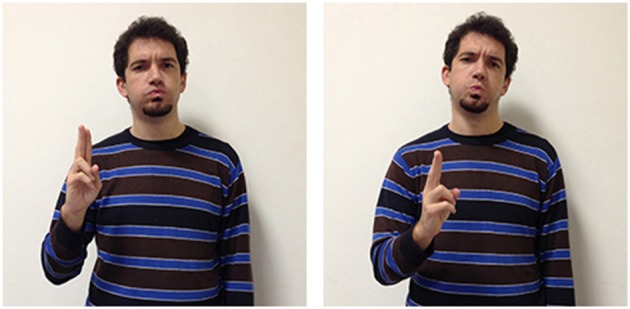
**LIS sign impossible_h-fff_**.

A variety of signs provide support for the theory according to which abstract terms refer more frequently to situations compared to concrete terms, which refer instead more often to objects and their properties. The three LIS signs in Figures 8, 9 highlight the importance of situations for concepts etymology and representation: they show that signs used in specific situations develop from signs used in similar situations and could all be glossed with the same English word impossible. These three signs, however, all have different forms, different origins, and are used in different sentences to express a slightly different meaning.

These three signs are examples of the phenomena of semantic change: signs that are initially grounded can become progressively more abstract and less transparent^3^ from an iconic perspective. The sign glossed as impossible_h-pa-pa_^4^, is probably derived from another sign, forbid, with which it shares the same handshape (extended index and middle fingers) and downward movement. In impossible_h-pa-pa_, however, the movement is repeated and more rapid. This form has assumed a more general meaning, allowing the signer to express the impossibility of an event or action, due to a decision taken from an authority, to the presence of unfavorable circumstances or to the absence of the necessary conditions for its implementation. The signer would use another sign glossed as impossible_h-fff_ in which the extended fingers move upward in a circular movement to categorically exclude the possibility that the conditions for an event to take place could exist. Wilcox et al. (2010) have proposed an interesting hypothesis on the origin of this LIS sign, which is relevant for us as it supports the idea that abstract words refer to events and situations. The sign impossible_h-fff_ seems to originate with the blessing gesture typical of Christian religion, and is similar to the gesture that has been historically reported to be used by speakers from the South of Italy to refer to a dead or dying person. It is worth noticing that this last variant has been incorporated into LIS as an autonomous lexical unit, i.e., the sign dead, produced without the mouth gesture “fff” which is co-produced in impossibile_h-fff._ The conceptual link between the blessing gesture and the sign expressing death is motivated by a metonymic contiguity, since priests are commonly required to bless dead people or people who are going to die. Given that death is associated to the preclusion of the possibility to live, it would have led metaphorically to the emergence of the extreme notion of impossibility expressed through the sign impossibile_h-fff_.

**Figure 10:**
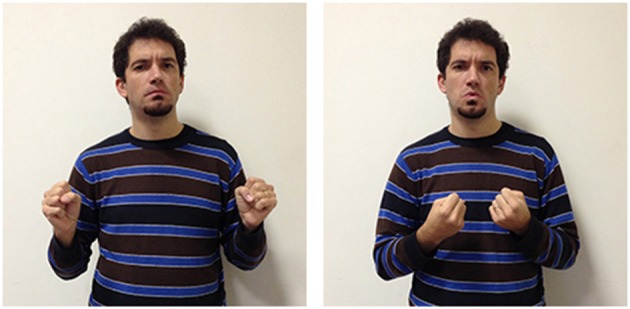
**LIS sign impossible_aaa_**.

**Figure 11:**
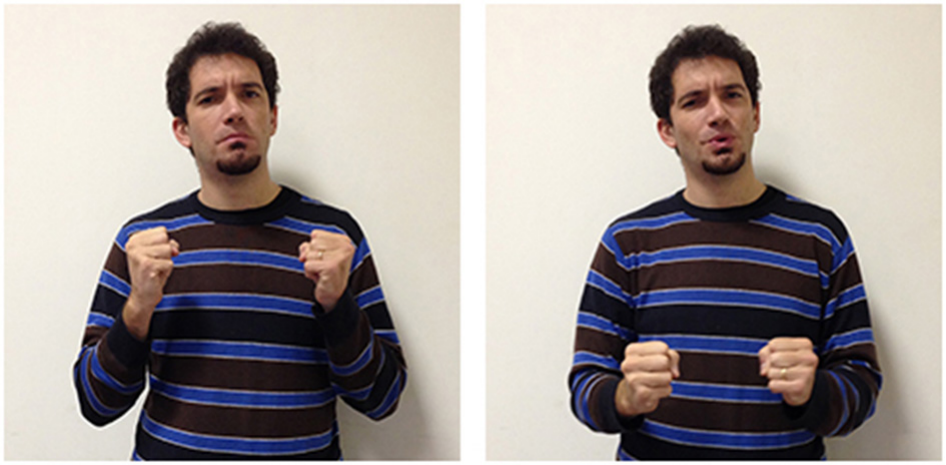
**LIS sign possible_aaa_**.

The third LIS sign, impossible_aa_ (Figure 10), has a semantic value that is less specific than the other two signs, as it expresses the notion that the conditions allowing a given action or event are absent, or that something cannot have given characteristics. This two-handed sign derives from the sign possible_aa_ (Figure 11), in which the signer expresses an evaluation on the existence of actual or potential conditions allowing an action or event. Both impossible_aa_ and possible_aa_ have the same hand configuration (two fists) but are performed with different movements. In possible_aa_ the two hands execute simultaneous repeated downward movements, while in impossible_aa_ the negation of a possibility is expressed through the alternate rotation of the forearms; this negation can be reinforced through a shaking head “no” movement. The close similarity between these two signs, possibile_aa_ and impossibile_aa_, illustrates how similarities and differences in the forms of signs are linked to semantic relations and/or oppositions (see Wilcox et al., 2010; Gianfreda et al., 2014).

**Figure 12:**
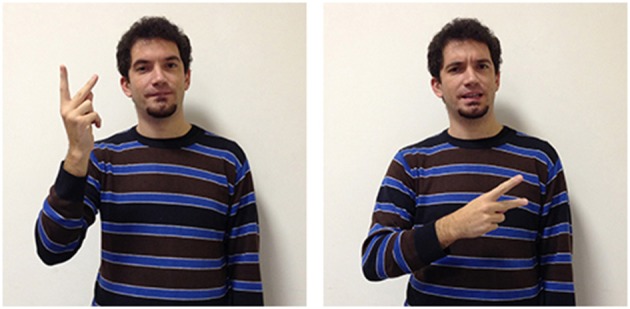
**LIS sign true**.

A different kind of situational conditioning is found in signs whose forms are influenced by the spoken or written language. For example, the LIS sign true (Figure 12) has a handshape which is also used for the letter V in the manual alphabet (extended index and middle fingers) and adds movement down and to the left of the face. This sign is typically used by signers, either to convey the idea that the described state of affair is true, or in order to clarify that the expressed position is valid.

The abstract meaning of “true” and “truth” is thus conveyed in LIS using a strategy known as “initialization.” In sign languages some signs are linked to the corresponding words through the use of a hand configuration which in the manual alphabet (used also in fingerspelling) represents the initial letter of the word having a corresponding meaning. In spoken/written Italian the corresponding words to the English words “true” and “truth” are “vero” and “verità,”both starting with the letter V. Other parameters of the lexical unit, such as movement and location, are not linked to the spoken/written language but are motivated by other factors. While LIS does not distinguish between “true” and “truth,” in ASL the two notions are represented differently. True is represented by using a sign grounded on the straight-path image schema (Roush, 2011), placing the dominant index finger against the signer's lips and then moving the finger forward several inches using a quick motion. So, the meaning of “true” is represented through the image of an object sent from the mouth along a straight line. In the nominalization form, truth, the sign is slightly varied in that the dominant hand with extended index and middle fingers move in a straight line to make contact with the open palm of the nondominant hand.

These examples help us understand how, in keeping with the WAT theory, the formation of abstract concepts can be influenced by multiple factors, some of which have linguistic origin.

These analyses show that the parameters of the sign's form can be motivated both by factors internal to the sign language as well as by the signers' relationship with another language having other characteristics, such as the spoken/written language.

A further example of how forms are influenced by other languages are seen in two other LIS signs. In Italian two different terms are used to distinguish the faculty for language (*linguaggio*) from a specific language used by a community of users (*lingua*) while in English the two concepts are labeled with the same term: “language.”

**Figure 13:**
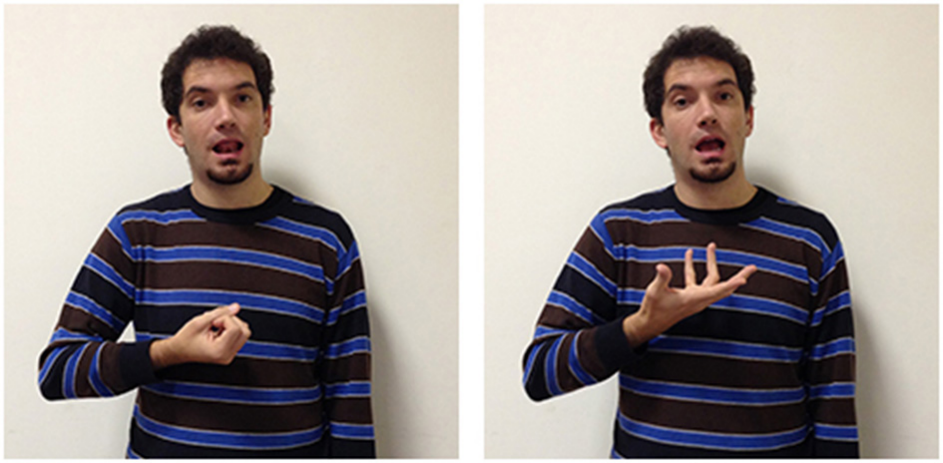
**LIS sign language/linguaggio**.

**Figure 14:**
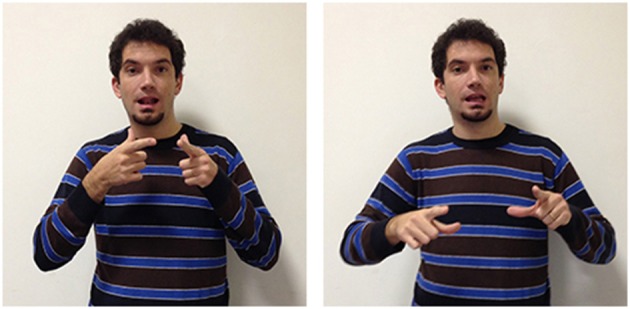
**LIS sign language/lingua**.

**Figure 15:**
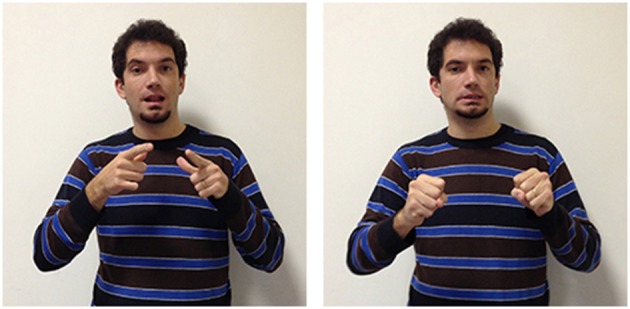
**LIS sign linguistics**.

These concepts are also differentiated by two different signs in LIS: in language/linguaggio (Figure 13) the hand moves up from the chest toward the external space and opens to a spread 5 handshape (very similar to the sign to express oneself); in language/lingua (Figure 14) both hands have an handshape associated with the letter “L” in the manual alphabet (extended index finger, thumb extended laterally). The hands, which are initially located in proximity of the mouth, move symmetrically forward with a wrist rotation. The sign linguistics (Figure 15) is very similar to the sign language/lingua, with the only exception that at the end of the movement the hands close into fists.

A final example is the LIS sign communication. This sign is similar to the ASL sign for the same concept: both hands have a handshape like the letter “C” in the manual alphabet and move forward and backward with a reciprocal alternate movement, possibly reflecting the underlying metaphor that “interaction is exchanging objects” (Roush, 2011). In LIS this sign has undergone interesting changes. In the past the sign was made in front of the mouth; now the sign is executed in the neutral space in front of the signer, perhaps related to a more recent cultural change in the concept resulting in communication not being conceived as being limited to spoken communication, but as also including manual and more general body communication.

All of the examples discussed above are interesting because they combine a strategy based on initialization with a process in which specific body parts (mouth, hand) and movements are involved to constrain and delimit the meaning.

## Conclusion

Our analyses and the examples provided are consistent with embodied and grounded theories of cognition, according to which abstract concepts are grounded in perception, action and emotional systems. What we find most important, however, is that sign languages can clarify the different kinds of grounding and thus contribute to the debate about how embodied theories can account for astractness. We considered and found examples supporting different kinds of embodied theories. The examples we made do not allow us to claim that a given theory is more valid compared to other theories. More systematic analyses would be necessary to advance such a claim. However, we think we are entitled to argue a. that an example can support or not a theory, or more than one theory; b. that, if the theory A is not able to explain a given sign which is rather explained by the theory B, the theory A cannot be considered as exhaustive.

We will discuss below what we consider the most important theoretical implications of the present work.

### Different levels of bodily involvement

First, our analysis indicates that, even if in sign languages the body is always involved to convey meanings, this involvement occurs at different levels. Skeptics of an embodied cognition perspective might object that it is not completely surprising that sign languages would provide evidence of grounding, given their visual nature and in particular the large amount of iconicity utilized by the language. In sign languages the coupling between language processing and sensori-motor processing becomes indeed more evident than in spoken languages. The body is always involved in spoken languages, for example through vocal articulators but in Sign languages the body, the hands and facial expressions become the main articulators. For example, the hands used for everyday activities such as pointing, enumerating or manipulating objects are also used for representing the same activities.

At the same time, however, it is possible to detect different levels of embodiment through a sign language analysis. The continuity between praxis, gesture and sign is easily recognizable at different levels of SLs structure: formational parameters, lexicon, morphology and syntax (see below for a more detailed discussion of this point). Despite this special characteristic of SLs has been widely recognized (e.g., Sandler and Lillo-Martin, 2006), only a few studies have explored the relationship between sign language and embodied theories, stressing the role of iconicity in sign languages (e.g., Pizzuto and Volterra, 2000; Boyes Braem et al., 2002; Morgan et al., 2008; Perniss et al., 2010). Iconicity can provide an additional mechanism for the grounding of language in sensorimotor systems; in SLs the presence of iconicity is pervasive, as a consequence SLs can be considered a special open window to better understand how language can be grounded. For example, according to Taub's (2001) cognitive-linguistic view, iconicity “is not an objective relationship between image and referent; rather, it is a relationship between our mental models of image and referent.” She claims that the creation of an iconic sign involves four successive stages: conceptualization, image selection, schematization, and sign encoding. The choice of the mental image is always mediated by cultural conventions, modality factors and language-specific conventions. This explains also why there is not an “Universal Sign Language” but rather many different Sign Languages. In a recent paper, Perniss and Vigliocco (2014) have highlighted the role of iconicity in both spoken and sign languages considering iconicity as a major vehicle for linking language and human sensory-motor experience. According to their perspective, iconicity represents the key to understand language evolution, development and processing providing a mechanism for displacement, referentiality and embodiment. They have also distinguished different types of iconic mapping, from a form of iconicity based more on imitative resemblance between the sign and the referent to a form of iconicity requiring more abstract mapping of features.

The novelty of our work, that recognizes the special and more evident role played by iconicity in Sign Languages, consists in focusing not only on the different levels of abstraction of the sign-referent mapping, but in identifying and examining a special case of referents, those of abstract concepts. Analyzing how signs can express abstract concepts in different ways (or through different iconic and not iconic mechanisms) provides some contributions to the debate on how different theories may account for abstract words representation. LIS can indeed provide interesting insights on the different degrees in which the various parameters of the signs are linked to the expressed concepts. In many cases specific locations assume an iconic meaning (for example, the majority of signs for mental activity are performed on the forehead), in other cases also the configuration and/or the movement performed are salient (for example, the sign clear is performed with an open hand configuration moving away from the eyes; a grasping movement characterizes the sign understand) (Pietrandrea, 2002).

### Support for the different embodied theories of abstract concepts

More crucially to the aim of the present paper, our work provides some insights and has a number of theoretical implications for the debate on how embodied and grounded theories might account for abstract concepts and words (see also Dove, 2009, 2011). The novelty of our work consists in investigating whether signs can provide support for the different embodied theories of abstract concepts.

In line with the previous literature on Conceptual Metaphor Theory, we found that many signs convey a metaphorical meaning and are based on underlying metaphors (e.g., the metaphors of knowing as seeing, of the head as container of mental activities, of the chest as container of feelings and emotions), in keeping with the view that abstract concepts are represented through a metaphorical mapping mechanism. However, in contrast with previous studies we have seen that this is not the whole story, for two main reasons.

The first is that our data support further embodied cognition theories according to which action, situations and emotions are important for abstract concepts representation. Some signs (e.g., the sign for impossible_h−fff_) provide evidence in favor of the view according to which abstract concepts are grounded on situations; other signs (e.g., the sign to constrain) offer support to the ACE view and other signs (e.g., the sign to express oneself) provide evidence favoring the emotion theory of abstract concepts. At a theoretical level the complex framework we obtained cast doubts on the possibility that a single explanation, for example based on a metaphorical mapping mechanism, is valid for the entire domain of abstract concepts and terms (See Prinz, 2002, 2012, for a similar view, according to which different abstract concepts can be explained referring to situation, to metaphors, to action as well as to linguistic information). At the same time, it confirms the necessity to perform fine-grained analyses of the differences between kinds of abstract concepts, analyses which some authors have started to conduct (e.g., Ghio et al., 2013; Roversi et al., 2013).

The second conclusion we can make is that, even if the analysis on LIS we performed provides support to all the aforementioned theories, at the same time it highlights their limitations. All these theories together are not able to fully account for the whole variety of signs we described. More importantly, they are not able to account for signs expressing some abstract concepts, such as truth.

We think that one of the main contributions of the present work consists in showing that, for some abstract concepts (e.g., the name of a discipline such as “linguistics,” a concept such as “truth,” etc.), LIS exploits linguistic information. This linguistic information could derive from different sources: from the same sign language (e.g., the LIS impossible_aa_ sign derives from the LIS sign possible_aa_), from a foreign sign language as ASL (e.g., language/lingua and linguistics) or from spoken/written Italian (e.g., true). This finding challenges many current embodied theories of abstract concepts and clearly supports the WAT view. More generally, it supports multiple representation views according to which not only sensorimotor but also emotional and especially linguistic information, differently distributed, characterize abstract concepts representation (beyond the WAT theory, see also Barsalou et al., 2008; Louwerse, 2011; see Kousta et al., 2011, for a multiple representation view stressing the role of emotions for abstract concepts and Dove, 2014, for a multiple representation view stressing the importance of language, similarly to WAT).

### A methodological note

Finally, a methodological note. LIS has proved to be an interesting and powerful mean to access how concepts are represented. We hope we have been able to suggest that the study of sign languages represents a fruitful and promising research line to investigate issues crucial for embodied and grounded cognition perspectives, in particular whether different degrees of embodiment exist (Taub, 2001) and whether they vary depending on the domain. Other studies have already demonstrated the importance of the study of sign languages for an embodied and grounded perspective. However, to our knowledge the present study is the first in which examples from a sign language are used to test and validate different theories on abstract concepts. Obviously a certain caution should be used, since, even though they are performed with the body, signs are, like words, arbitrary, so it is difficult to argue that they reflect directly the way concepts are represented. However, they are surely more grounded and to a certain extent more “visible” than words, thus they certainly represent an important cue to help understand conceptual representation. The present paper, being a theoretical paper rather than an experimental one, intends to indicate a possible direction of work. In order to perform a more systematic and thorough analysis, one would need to ask LIS signers to rate different kinds of signs in terms of abstractness, and then select a subset of signs evaluated as abstract and analyze them. Future work is planned to perform such an analysis.

Overall, we think our work provides some hints for how to address issues related to the future of embodied cognition and to the notion of body. Our LIS analyses suggest that, even if the signs we described always involve the body, different degrees of embodiment might be present. Furthermore, our results suggest that to account for abstract concepts not only sensorimotor and emotional experience should be called into play, but that also linguistic information plays a major role. This might appear in conflict with an embodied approach. We believe it is not, since language is not a disembodied activity but an important part of our total human experience. A challenge for future research is to identify sub-sets of abstract concepts, and to determine whether linguistic information becomes progressively more relevant, the higher the degree of concepts abstractness is.

### Conflict of interest statement

The authors declare that the research was conducted in the absence of any commercial or financial relationships that could be construed as a potential conflict of interest.

## Footnotes

^1^Glosses, better known as interlinear glosses, are used in different areas of linguistics in order to give an account of the meaning/description of the morphemes of a given language. The use of glosses in sign language research is a useful practice, but should not be considered a self sufficient representation system neglecting the general requirement of being associated with a transcription of the form of the morpheme. Otherwise it is not possible to verify (discuss or contradict) any morphological analysis conducted, since no formal property of the sign can be used in order to check the consistency of data and analysis provided (Pizzuto and Pietrandrea, 2001; Petitta et al., 2013).

^2^In LIS, as in all sign languages analyzed sofar, signs are often accompanied by mouth patterns. Two main categories are distinguished: (i) mouthings which are derived and represent words or parts of words from a spoken language, and (ii) mouth gestures which are idiomatic gestures produced by the mouth not related to a spoken language (Boyes Braem and Sutton Spence, 2001; Fontana, 2008).

^3^Research on iconicity has traditionally distinguishes between transparent (the meaning can be guessed by everyone), translucent (a non-signer can choose among alternative the right ones, once the meaning is known) and opaque (no iconically motivated link can be found) signs (Bellugi and Klima, 1976; Klima and Bellugi, 1979; Pizzuto and Volterra, 2000; Perniss and Vigliocco, 2014).

^4^The letter “H” reported in subscript is conventionally used because this handshape represents the letter H in the manual alphabet. The symbol “pa-pa” refers to the mouth gesture obligatorily requested in the sign execution.
