# Supplementary figures and images for: The body and the fading away of abstract concepts and words: a sign language analysis
Source: Front Psychol. 2014 Jul 29;5:811. doi: 10.3389/fpsyg.2014.00811 (PMC4114187; doi:10.3389/fpsyg.2014.00811)

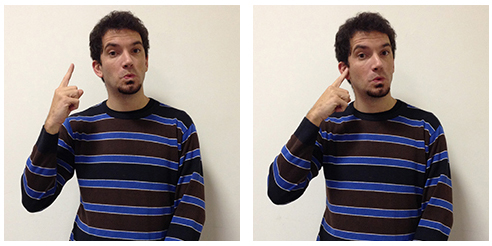

Supplement: Supplementary file 1 [file DataSheet1.ZIP › 17 TO HEAR.jpg]

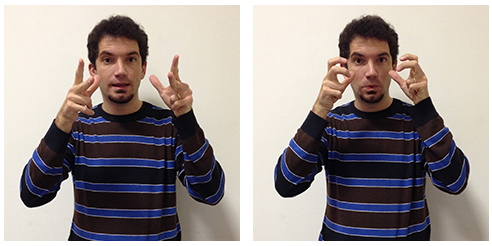

Supplement: Supplementary file 1 [file DataSheet1.ZIP › 18 PERCEIVE-THROUGH-SIGHT.jpg]

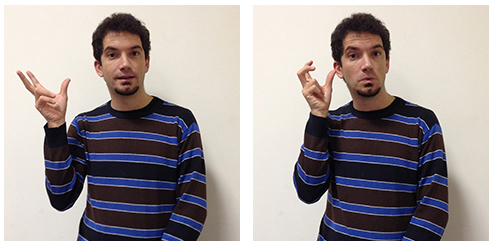

Supplement: Supplementary file 1 [file DataSheet1.ZIP › 19 PERCEIVE-THROUGH-HEARING.jpg]

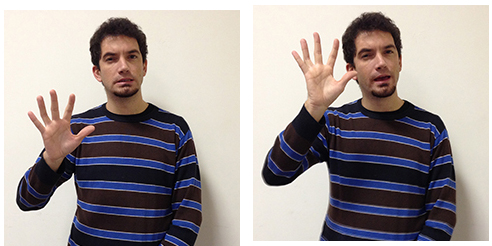

Supplement: Supplementary file 1 [file DataSheet1.ZIP › 20 SEEM.jpg]

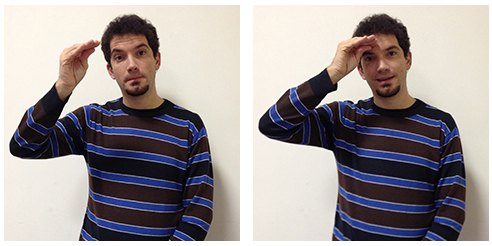

Supplement: Supplementary file 1 [file DataSheet1.ZIP › 21 TO LEARN.jpg]

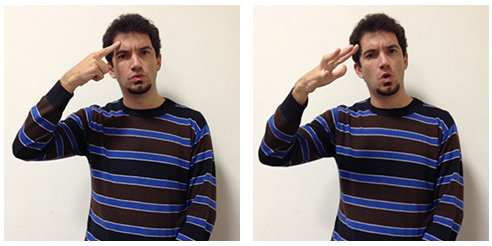

Supplement: Supplementary file 1 [file DataSheet1.ZIP › 22 ACKNOWLEDGED.jpg]

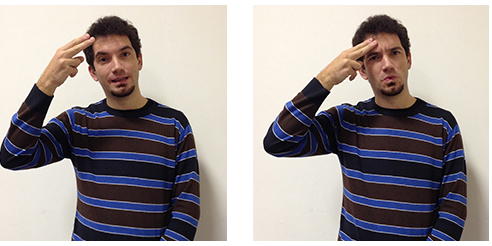

Supplement: Supplementary file 1 [file DataSheet1.ZIP › 23 TO REMEMBER.jpg]

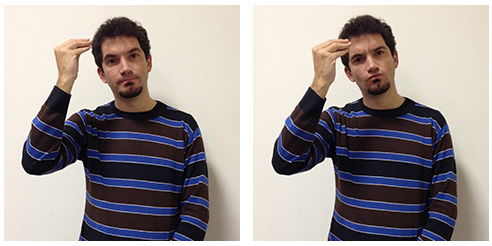

Supplement: Supplementary file 1 [file DataSheet1.ZIP › 24 TO THINK.jpg]

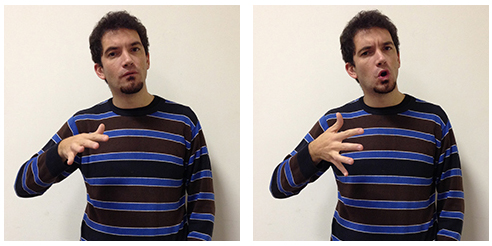

Supplement: Supplementary file 1 [file DataSheet1.ZIP › 25 MAYBE.jpg]

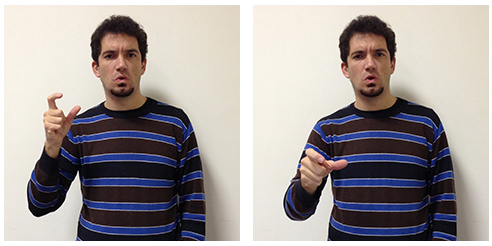

Supplement: Supplementary file 1 [file DataSheet1.ZIP › 26 TO CONSTRAIN.jpg]

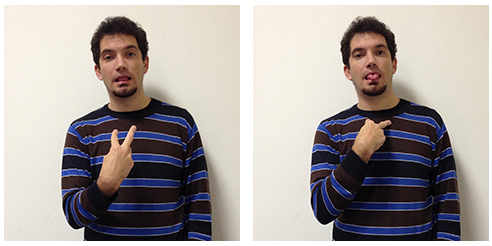

Supplement: Supplementary file 1 [file DataSheet1.ZIP › 27 TO BE CONSTRAIN.jpg]

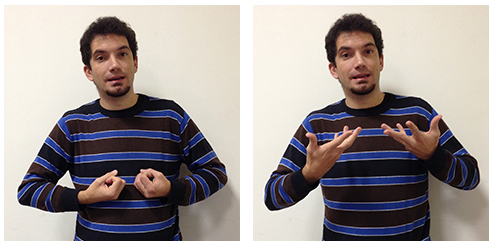

Supplement: Supplementary file 1 [file DataSheet1.ZIP › 28 TO EXPRESS ONESELF.jpg]

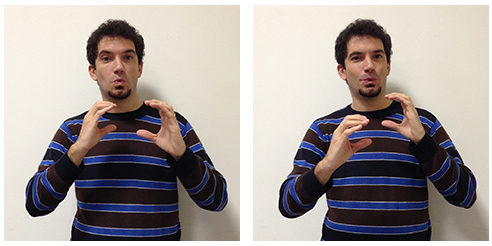

Supplement: Supplementary file 1 [file DataSheet1.ZIP › 29 COMMUNICATION.jpg]

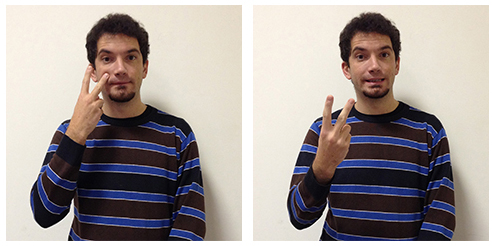

Supplement: Supplementary file 1 [file DataSheet1.ZIP › 16 TO SEE.jpg]
